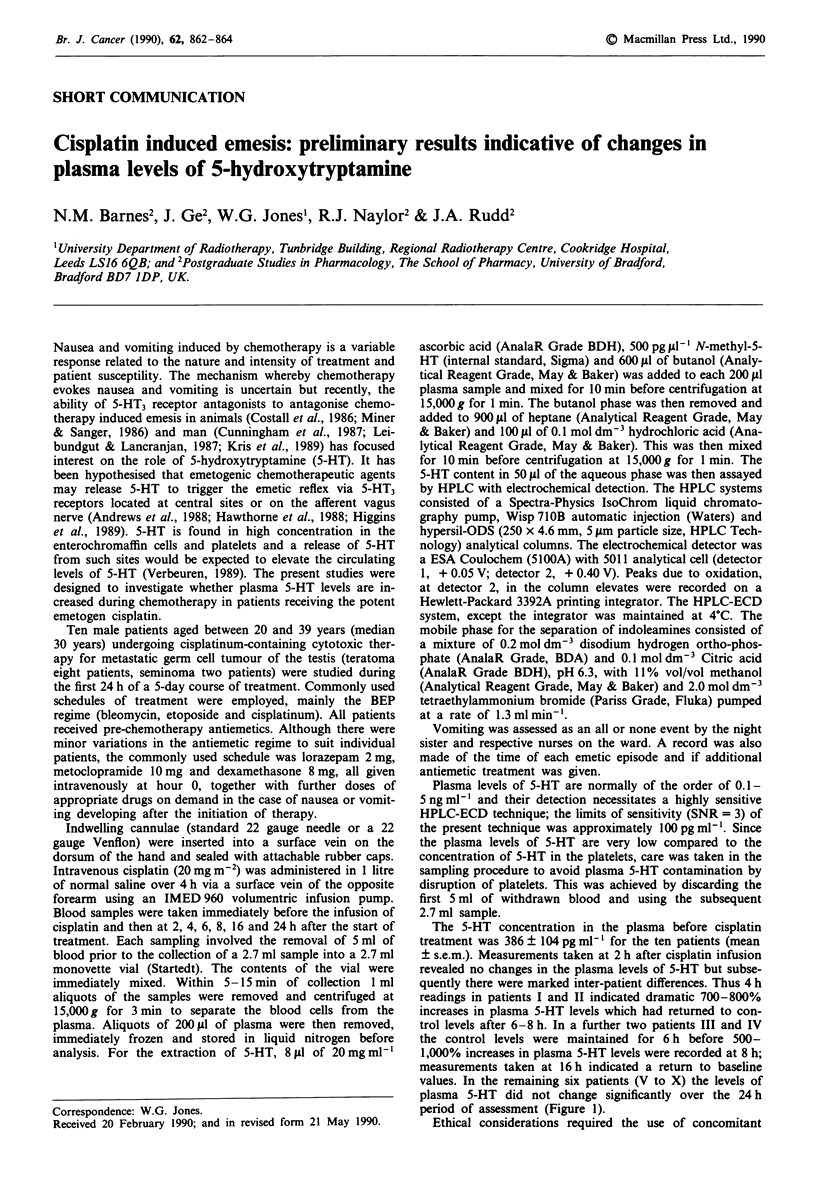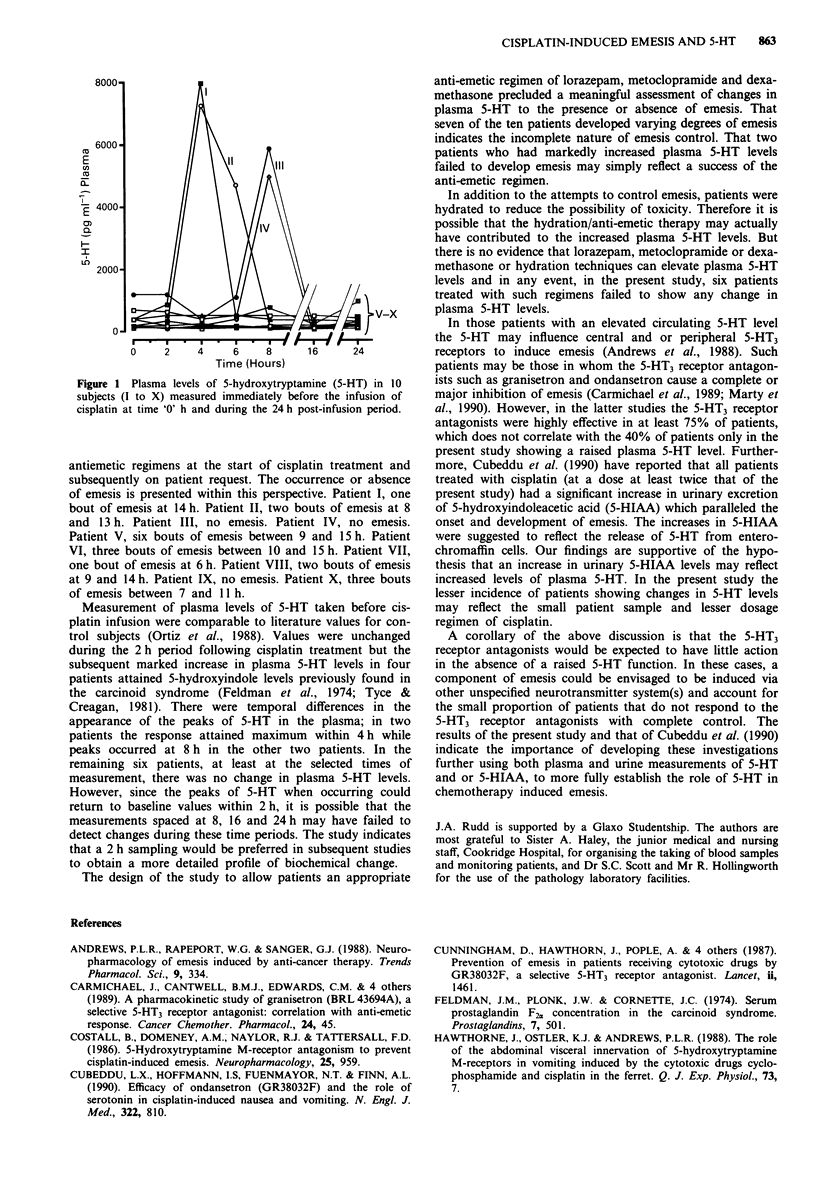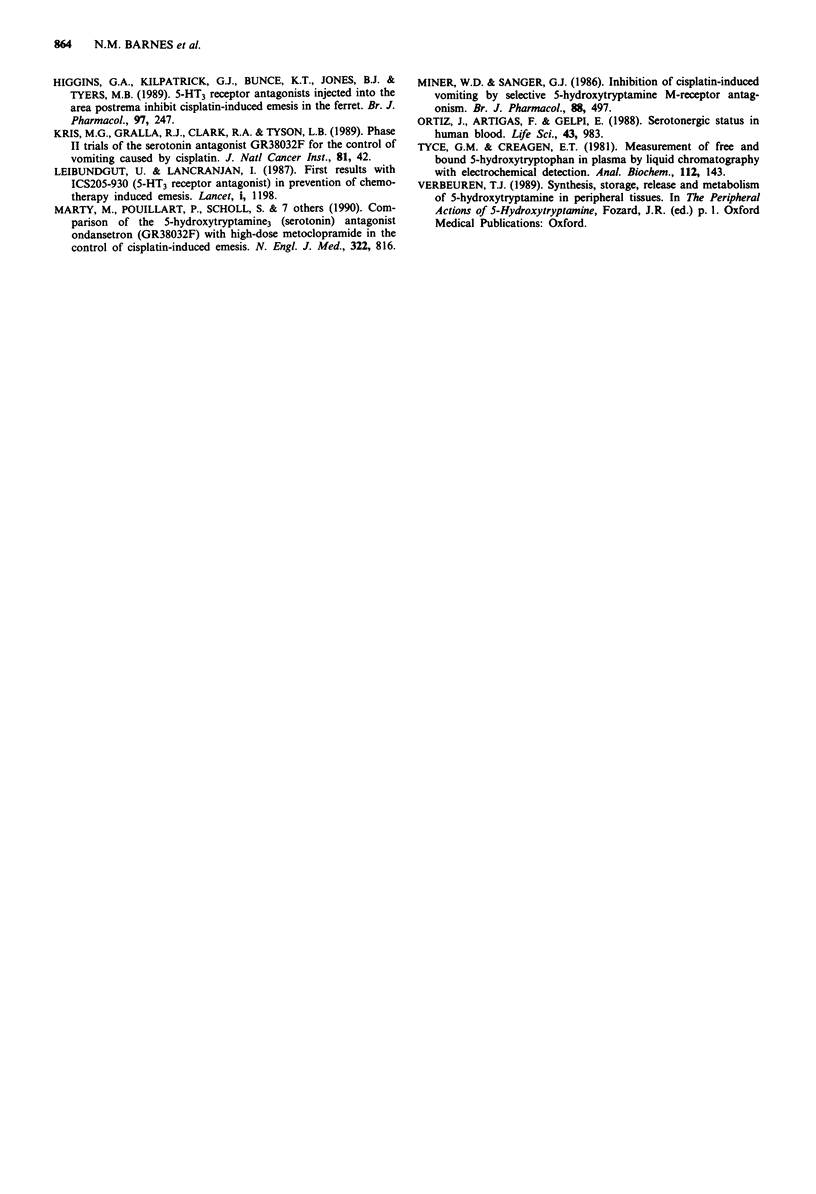# Cisplatin induced emesis: preliminary results indicative of changes in plasma levels of 5-hydroxytryptamine.

**DOI:** 10.1038/bjc.1990.395

**Published:** 1990-11

**Authors:** N. M. Barnes, J. Ge, W. G. Jones, R. J. Naylor, J. A. Rudd

**Affiliations:** University Department of Radiotherapy, Cookridge Hospital, Leeds, UK.


Br. J. Cancer (1990), 62, 862-864 © Macmillan Press Ltd., 1990

SHORT COMMUNICATION

### Cisplatin induced emesis: preliminary results indicative of changes in plasma levels of 5-hydroxytryptamine

N.M. Barnes[2], J. Ge[2], W.G. Jones[1], R.J. Naylor[2] & J.A. Rudd[2]

[1]*University Department of Radiotherapy, Tunbridge Building, Regional Radiotherapy Centre, Cookridge Hospital, Leeds LS16 6QB; and* [2]*Postgraduate Studies in Pharmacology, The School of Pharmacy, University of Bradford, Bradford BD7 1DP, UK.*

Nausea and vomiting induced by chemotherapy is a variable response related to the nature and intensity of treatment and patient susceptility. The mechanism whereby chemotherapy evokes nausea and vomiting is uncertain but recently, the ability of 5-$HT_3$ receptor antagonists to antagonise chemotherapy induced emesis in animals (Costall *et al.*, 1986; Miner & Sanger, 1986) and man (Cunningham *et al.*, 1987; Leibundgut & Lancranjan, 1987; Kris *et al.*, 1989) has focused interest on the role of 5-hydroxytryptamine (5-HT). It has been hypothesised that emetogenic chemotherapeutic agents may release 5-HT to trigger the emetic reflex via 5-$HT_3$ receptors located at central sites or on the afferent vagus nerve (Andrews *et al.*, 1988; Hawthorne *et al.*, 1988; Higgins *et al.*, 1989). 5-HT is found in high concentration in the enterochromaffin cells and platelets and a release of 5-HT from such sites would be expected to elevate the circulating levels of 5-HT (Verbeuren, 1989). The present studies were designed to investigate whether plasma 5-HT levels are increased during chemotherapy in patients receiving the potent emetogen cisplatin.

Ten male patients aged between 20 and 39 years (median 30 years) undergoing cisplatinum-containing cytotoxic therapy for metastatic germ cell tumour of the testis (teratoma eight patients, seminoma two patients) were studied during the first 24 h of a 5-day course of treatment. Commonly used schedules of treatment were employed, mainly the BEP regime (bleomycin, etoposide and cisplatinum). All patients received pre-chemotherapy antiemetics. Although there were minor variations in the antiemetic regime to suit individual patients, the commonly used schedule was lorazepam 2 mg, metoclopramide 10 mg and dexamethasone 8 mg, all given intravenously at hour 0, together with further doses of appropriate drugs on demand in the case of nausea or vomiting developing after the initiation of therapy.

Indwelling cannulae (standard 22 gauge needle or a 22 gauge Venflon) were inserted into a surface vein on the dorsum of the hand and sealed with attachable rubber caps. Intravenous cisplatin (20 mg $m^{-2}$) was administered in 1 litre of normal saline over 4 h via a surface vein of the opposite forearm using an IMED 960 volumentric infusion pump. Blood samples were taken immediately before the infusion of cisplatin and then at 2, 4, 6, 8, 16 and 24 h after the start of treatment. Each sampling involved the removal of 5 ml of blood prior to the collection of a 2.7 ml sample into a 2.7 ml monovette vial (Startedt). The contents of the vial were immediately mixed. Within 5-15 min of collection 1 ml aliquots of the samples were removed and centrifuged at 15,000 *g* for 3 min to separate the blood cells from the plasma. Aliquots of 200 µl of plasma were then removed, immediately frozen and stored in liquid nitrogen before analysis. For the extraction of 5-HT, 8 µl of 20 mg $ml^{-1}$ ascorbic acid (AnalaR Grade BDH), 500 pg $\mu l^{-1}$ *N*-methyl-5-HT (internal standard, Sigma) and 600 µl of butanol (Analytical Reagent Grade, May & Baker) was added to each 200 µl plasma sample and mixed for 10 min before centrifugation at 15,000 *g* for 1 min. The butanol phase was then removed and added to 900 µl of heptane (Analytical Reagent Grade, May & Baker) and 100 µl of 0.1 mol $dm^{-3}$ hydrochloric acid (Analytical Reagent Grade, May & Baker). This was then mixed for 10 min before centrifugation at 15,000 *g* for 1 min. The 5-HT content in 50 µl of the aqueous phase was then assayed by HPLC with electrochemical detection. The HPLC systems consisted of a Spectra-Physics IsoChrom liquid chromatography pump, Wisp 710B automatic injection (Waters) and hypersil-ODS (250 × 4.6 mm, 5 µm particle size, HPLC Technology) analytical columns. The electrochemical detector was a ESA Coulochem (5100A) with 5011 analytical cell (detector 1, + 0.05 V; detector 2, + 0.40 V). Peaks due to oxidation, at detector 2, in the column elevates were recorded on a Hewlett-Packard 3392A printing integrator. The HPLC-ECD system, except the integrator was maintained at 4°C. The mobile phase for the separation of indoleamines consisted of a mixture of 0.2 mol $dm^{-3}$ disodium hydrogen ortho-phosphate (AnalaR Grade, BDA) and 0.1 mol $dm^{-3}$ Citric acid (AnalaR Grade BDH), pH 6.3, with 11% vol/vol methanol (Analytical Reagent Grade, May & Baker) and 2.0 mol $dm^{-3}$ tetraethylammonium bromide (Pariss Grade, Fluka) pumped at a rate of 1.3 ml $min^{-1}$.

Vomiting was assessed as an all or none event by the night sister and respective nurses on the ward. A record was also made of the time of each emetic episode and if additional antiemetic treatment was given.

Plasma levels of 5-HT are normally of the order of 0.1-5 ng $ml^{-1}$ and their detection necessitates a highly sensitive HPLC-ECD technique; the limits of sensitivity (SNR = 3) of the present technique was approximately 100 pg $ml^{-1}$. Since the plasma levels of 5-HT are very low compared to the concentration of 5-HT in the platelets, care was taken in the sampling procedure to avoid plasma 5-HT contamination by disruption of platelets. This was achieved by discarding the first 5 ml of withdrawn blood and using the subsequent 2.7 ml sample.

The 5-HT concentration in the plasma before cisplatin treatment was 386 ± 104 pg $ml^{-1}$ for the ten patients (mean ± s.e.m.). Measurements taken at 2 h after cisplatin infusion revealed no changes in the plasma levels of 5-HT but subsequently there were marked inter-patient differences. Thus 4 h readings in patients I and II indicated dramatic 700-800% increases in plasma 5-HT levels which had returned to control levels after 6-8 h. In a further two patients III and IV the control levels were maintained for 6 h before 500-1,000% increases in plasma 5-HT levels were recorded at 8 h; measurements taken at 16 h indicated a return to baseline values. In the remaining six patients (V to X) the levels of plasma 5-HT did not change significantly over the 24 h period of assessment (Figure 1).

Ethical considerations required the use of concomitant

Correspondence: W.G. Jones.
Received 20 February 1990; and in revised form 21 May 1990.

**Figure 1** Plasma levels of 5-hydroxytryptamine (5-HT) in 10 subjects (I to X) measured immediately before the infusion of cisplatin at time '0' h and during the 24 h post-infusion period.

antiemetic regimens at the start of cisplatin treatment and subsequently on patient request. The occurrence or absence of emesis is presented within this perspective. Patient I, one bout of emesis at 14 h. Patient II, two bouts of emesis at 8 and 13 h. Patient III, no emesis. Patient IV, no emesis. Patient V, six bouts of emesis between 9 and 15 h. Patient VI, three bouts of emesis between 10 and 15 h. Patient VII, one bout of emesis at 6 h. Patient VIII, two bouts of emesis at 9 and 14 h. Patient IX, no emesis. Patient X, three bouts of emesis between 7 and 11 h.

Measurement of plasma levels of 5-HT taken before cisplatin infusion were comparable to literature values for control subjects (Ortiz *et al.*, 1988). Values were unchanged during the 2 h period following cisplatin treatment but the subsequent marked increase in plasma 5-HT levels in four patients attained 5-hydroxyindole levels previously found in the carcinoid syndrome (Feldman *et al.*, 1974; Tyce & Creagan, 1981). There were temporal differences in the appearance of the peaks of 5-HT in the plasma; in two patients the response attained maximum within 4 h while peaks occurred at 8 h in the other two patients. In the remaining six patients, at least at the selected times of measurement, there was no change in plasma 5-HT levels. However, since the peaks of 5-HT when occurring could return to baseline values within 2 h, it is possible that the measurements spaced at 8, 16 and 24 h may have failed to detect changes during these time periods. The study indicates that a 2 h sampling would be preferred in subsequent studies to obtain a more detailed profile of biochemical change.

The design of the study to allow patients an appropriate anti-emetic regimen of lorazepam, metoclopramide and dexamethasone precluded a meaningful assessment of changes in plasma 5-HT to the presence or absence of emesis. That seven of the ten patients developed varying degrees of emesis indicates the incomplete nature of emesis control. That two patients who had markedly increased plasma 5-HT levels failed to develop emesis may simply reflect a success of the anti-emetic regimen.

In addition to the attempts to control emesis, patients were hydrated to reduce the possibility of toxicity. Therefore it is possible that the hydration/anti-emetic therapy may actually have contributed to the increased plasma 5-HT levels. But there is no evidence that lorazepam, metoclopramide or dexamethasone or hydration techniques can elevate plasma 5-HT levels and in any event, in the present study, six patients treated with such regimens failed to show any change in plasma 5-HT levels.

In those patients with an elevated circulating 5-HT level the 5-HT may influence central and or peripheral 5-$HT_3$ receptors to induce emesis (Andrews *et al.*, 1988). Such patients may be those in whom the 5-$HT_3$ receptor antagonists such as granisetron and ondansetron cause a complete or major inhibition of emesis (Carmichael *et al.*, 1989; Marty *et al.*, 1990). However, in the latter studies the 5-$HT_3$ receptor antagonists were highly effective in at least 75% of patients, which does not correlate with the 40% of patients only in the present study showing a raised plasma 5-HT level. Furthermore, Cubeddu *et al.* (1990) have reported that all patients treated with cisplatin (at a dose at least twice that of the present study) had a significant increase in urinary excretion of 5-hydroxyindoleacetic acid (5-HIAA) which paralleled the onset and development of emesis. The increases in 5-HIAA were suggested to reflect the release of 5-HT from enterochromaffin cells. Our findings are supportive of the hypothesis that an increase in urinary 5-HIAA levels may reflect increased levels of plasma 5-HT. In the present study the lesser incidence of patients showing changes in 5-HT levels may reflect the small patient sample and lesser dosage regimen of cisplatin.

A corollary of the above discussion is that the 5-$HT_3$ receptor antagonists would be expected to have little action in the absence of a raised 5-HT function. In these cases, a component of emesis could be envisaged to be induced via other unspecified neurotransmitter system(s) and account for the small proportion of patients that do not respond to the 5-$HT_3$ receptor antagonists with complete control. The results of the present study and that of Cubeddu *et al.* (1990) indicate the importance of developing these investigations further using both plasma and urine measurements of 5-HT and or 5-HIAA, to more fully establish the role of 5-HT in chemotherapy induced emesis.

J.A. Rudd is supported by a Glaxo Studentship. The authors are most grateful to Sister A. Haley, the junior medical and nursing staff, Cookridge Hospital, for organising the taking of blood samples and monitoring patients, and Dr S.C. Scott and Mr R. Hollingworth for the use of the pathology laboratory facilities.

### References

ANDREWS, P.L.R., RAPEPORT, W.G. & SANGER, G.J. (1988). Neuropharmacology of emesis induced by anti-cancer therapy. *Trends Pharmacol. Sci.*, **9**, 334.

CARMICHAEL, J., CANTWELL, B.M.J., EDWARDS, C.M. & 4 others (1989). A pharmacokinetic study of granisetron (BRL 43694A), a selective 5-$HT_3$ receptor antagonist: correlation with anti-emetic response. *Cancer Chemother. Pharmacol.*, **24**, 45.

COSTALL, B., DOMENEY, A.M., NAYLOR, R.J. & TATTERSALL, F.D. (1986). 5-Hydroxytryptamine M-receptor antagonism to prevent cisplatin-induced emesis. *Neuropharmacology*, **25**, 959.

CUBEDDU, L.X., HOFFMANN, I.S, FUENMAYOR, N.T. & FINN, A.L. (1990). Efficacy of ondansetron (GR38032F) and the role of serotonin in cisplatin-induced nausea and vomiting. *N. Engl. J. Med.*, **322**, 810.

CUNNINGHAM, D., HAWTHORN, J., POPLE, A. & 4 others (1987). Prevention of emesis in patients receiving cytotoxic drugs by GR38032F, a selective 5-$HT_3$ receptor antagonist. *Lancet*, **ii**, 1461.

FELDMAN, J.M., PLONK, J.W. & CORNETTE, J.C. (1974). Serum prostaglandin $F_{2\alpha}$ concentration in the carcinoid syndrome. *Prostaglandins*, **7**, 501.

HAWTHORNE, J., OSTLER, K.J. & ANDREWS, P.L.R. (1988). The role of the abdominal visceral innervation of 5-hydroxytryptamine M-receptors in vomiting induced by the cytotoxic drugs cyclophosphamide and cisplatin in the ferret. *Q. J. Exp. Physiol.*, **73**, 7.

HIGGINS, G.A., KILPATRICK, G.J., BUNCE, K.T., JONES, B.J. & TYERS, M.B. (1989). 5-$HT_3$ receptor antagonists injected into the area postrema inhibit cisplatin-induced emesis in the ferret. *Br. J. Pharmacol.*, **97**, 247.

KRIS, M.G., GRALLA, R.J., CLARK, R.A. & TYSON, L.B. (1989). Phase II trials of the serotonin antagonist GR38032F for the control of vomiting caused by cisplatin. *J. Natl Cancer Inst.*, **81**, 42.

LEIBUNDGUT, U. & LANCRANJAN, I. (1987). First results with ICS205-930 (5-$HT_3$ receptor antagonist) in prevention of chemotherapy induced emesis. *Lancet*, **i**, 1198.

MARTY, M., POUILLART, P., SCHOLL, S. & 7 others (1990). Comparison of the 5-hydroxytryptamine$_3$ (serotonin) antagonist ondansetron (GR38032F) with high-dose metoclopramide in the control of cisplatin-induced emesis. *N. Engl. J. Med.*, **322**, 816.

MINER, W.D. & SANGER, G.J. (1986). Inhibition of cisplatin-induced vomiting by selective 5-hydroxytryptamine M-receptor antagonism. *Br. J. Pharmacol.*, **88**, 497.

ORTIZ, J., ARTIGAS, F. & GELPI, E. (1988). Serotonergic status in human blood. *Life Sci.*, **43**, 983.

TYCE, G.M. & CREAGEN, E.T. (1981). Measurement of free and bound 5-hydroxytryptophan in plasma by liquid chromatography with electrochemical detection. *Anal. Biochem.*, **112**, 143.

VERBEUREN, T.J. (1989). Synthesis, storage, release and metabolism of 5-hydroxytryptamine in peripheral tissues. In *The Peripheral Actions of 5-Hydroxytryptamine*, Fozard, J.R. (ed.) p. 1. Oxford Medical Publications: Oxford.